# On-site colorimetric detection of *Salmonella typhimurium*

**DOI:** 10.1038/s41538-022-00164-0

**Published:** 2022-10-17

**Authors:** Shengnan Wei, Zhenyue Su, Xiangong Bu, Xuening Shi, Bo Pang, Liang Zhang, Juan Li, Chao Zhao

**Affiliations:** School of Public Health, Jilin University, Changchun, Jilin 130021 China

**Keywords:** Nanoparticles, Biosensors

## Abstract

Rapid qualitative and quantitative detection of *Salmonella typhimurium* (*S. typhimurium*) takes an important role in ensuring food safety. Herein, a colorimetric assay aptasensor for *S. typhimurium* utilizing intrinsic peroxidase-like activity of gold nanoparticles embedded spherical covalent organic framework and the affinity and specificity of *S. typhimurium*-aptamer has been explored. This aptasensor can capture the *S. typhimurium* via the selective binding effect of aptamer, and the catalytically active sites were shielded. As a result, the colorimetric signals of the 3,3′,5,5′-tetramethylbenzidine-H_2_O_2_ system were turned off. Under optimum conditions, the aptasensor gave a linear response over the range of 10 to 10^7^ CFU/mL for *S. typhimurium*. The detection limit of 7 CFU/mL was obtained within 45 min and was effectively applied to detect *S. typhimurium* in milk and lake water samples with recoveries in the range from 96.4 to 101.0%. More importantly, combined with a self-developed smartphone-based image analysis system, the proposed aptasensor can be used for point-of-care testing applications.

## Introduction

*Salmonella*, as one of the dominating biological factors of foodborne diseases, poses a risk to human health^1^. According to the World Health Organization (WHO) data in 2021, there were 58 food safety incidents caused by *Salmonella* in 86 of the WHO Member States and territories^2^. The incidence rate of *Salmonella typhimurium* (*S. typhimurium*) ranks first among *Salmonella* infections and *S. typhimurium* can cause nosocomial infection and fulminant food poisoning with a high fatality rate^3^. Sensitive and accurate identification and detection of *S. typhimurium* are important for early prevention, diagnosis, and control of foodborne disease infections and outbreaks. Therefore, the detection technology of *S. typhimurium* is a significant research hotspot in public health. Traditional detection methods for *S. typhimurium* include plating culture^4^, enzyme-linked immunosorbent assays (ELISA)^5^, and polymerase chain reaction (PCR)^6^. Although these techniques are used as gold standards or recommended detection methods, it is still essential to construct more convenient, easily operable, and accurate assay methods to meet the demand of on-site testing of *S. typhimurium*. In the past few years, various novel biosensors have been widely developed, such as colorimetric^7^, fluorescence^8^, surface-enhanced Raman scattering (SERS)^9^, and electrochemical^10^ biosensors. They have the merits of rapid response, easy to use, low cost, good sensitivity, and selectivity. Colorimetric biosensors can be used to detect analytes through color changes directly by naked eyes or portable optical equipment for measurement^11^. Furthermore, the colorimetric assay is very suitable to be combined with smartphone-based systems to provide qualitative and semi-quantitative analysis services for field detection. The colorimetric methods mainly include two types: one is based on the optical properties of the probe itself to produce colorimetric signals, and the other is based on the color change of the chromogenic substrate via enzymatic catalysis reactions^12^. The former is susceptible to interference from impurities in the food samples to affect accuracy. For example, gold nanoparticles (AuNPs) can present color change from red to blue when transform from a monodispersed state to an aggregated state in liquid solution. However, bare AuNPs self-aggregation easily occurs due to the passivating surface layer, ionic strength, pH, and temperature resulting in associated color change^13,14^. For example, some colorimetric assays were established based on the aggregation of AuNPs by metal ion inducers, which might make results prone to false positives^15,16^. Therefore, the colorimetric method based on enzymatic catalysis is a good way and it has been rapidly developed in recent years^7^. Due to the poor stability and expense of natural enzymes, more attention has been paid to nanozyme as biosensors in colorimetric assay systems, especially nanozyme mimicking peroxidase has been widely reported^17^. At present, numerous nanomaterials with peroxidase-like activity have been explored, such as noble metal NPs (Au, Ag, Pt), manganese dioxide NPs and carbon-based nanomaterials (graphene oxide (GO))^18,19^. AuNPs have excellent intrinsic peroxidase-like activity and they could be labeled by biological molecules easily for different applications^19,20^. The catalytic activity of AuNPs is susceptible to interference by various factors, including shape, size, surface charge, and surface coating residues^21^. For example, smaller AuNPs generally have better catalytic performance due to a higher population of low-coordinated gold atoms. Citrate as a surface coating residue of AuNPs can negatively affect the peroxidase-like activity of AuNPs. In addition, external parameters including temperature and pH play an important role in the catalytic performance^22^. Therefore, we need to solve the problem of keeping good dispersity and ensuring that the catalytic activity is not affected in the process of detecting the target in various complex samples.

Covalent organic framework (COF) is a kind of promising porous polymeric material with a large specific surface area, adjustable pore structure, and high stability^23^. COFs have been widely designed and employed for gas storage and separation, drug encapsulation, ionic-exchange processes, photoconductivity, catalysis, chemical sensing, and contaminants for residual water treatment^23–25^. The ideal and controllable skeleton structure of the COFs could be employed in supporting some metal nanoparticles such as PdNPs, PtNPs, and AuNPs to develop novel COF-metal NPs composites^25–27^. Electrochemical, fluorescence, electrochemiluminescence, and other biosensing strategies based on these COF-metal NPs composites have been reported^28–30^. In 2019, Li et al. reported a COF with sheet structure obtained by 1,3,5- Tris-(4-formyl-phenyl) triazine (PT) and 4, 4′-azodianiline (Azo), then modified AuNPs on this COF to form COF (PTAzo)-AuNPs. The peroxidase-like activity of COF (PTAzo)-AuNPs is very low^31^. Compared with this sheet structure, the COF with a spherical structure could have a larger specific surface area to dope more AuNPs (COF-AuNPs), so the peroxidase-like activity of COF-AuNPs is expected to be improved. At present, there is no study focused on using spherical COF as the skeleton to enhance the peroxidase-like activity and stability of AuNPs.

**Fig. 1:**
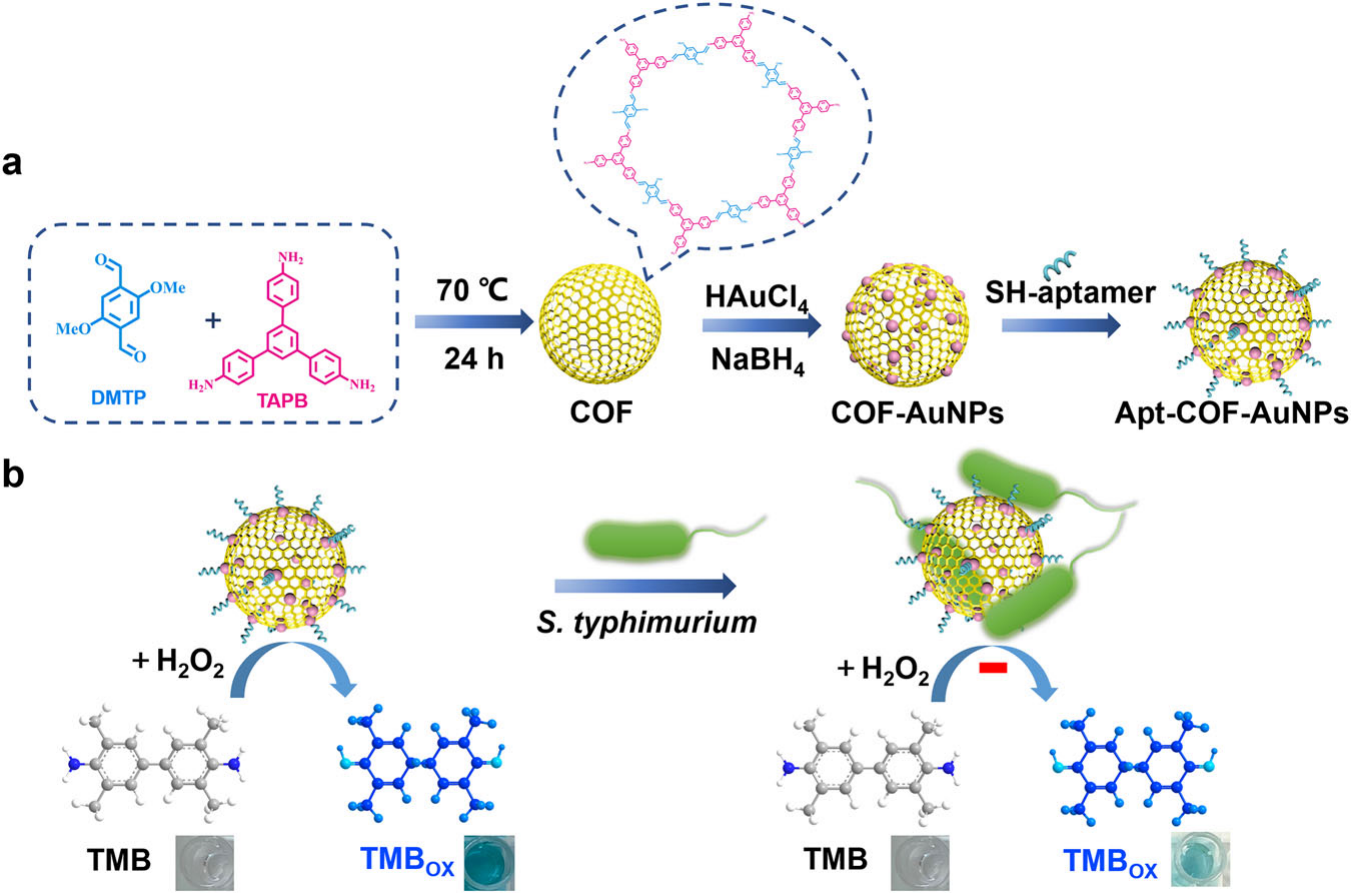
Schematic representation of the assay for detection of *S. typhimurium*. **a** The preparation technology for apt-COF-AuNPs and **b** Colorimetric detection of *S. typhimurium*. *S. typhimurium*-inhibited peroxidase activity of apt-COF-AuNPs slowed down the oxidation of TMB with H_2_O_2_, giving the samples a lighter blue color. All images created by authors.

Inspired by the above considerations, we used the COF with the spherical structure as a frame for loading AuNPs and constructed a simple and rapid colorimetric biosensor to detect *S. typhimurium* based on the peroxidase-like activity of COF-AuNPs. The entire process of the proposed colorimetric assay method for *S. typhimurium* was illustrated in Fig. 1. COF-AuNPs were prepared using 1,3,5-tris(4-aminophenyl) benzene (TAPB) and 2,5-dimethoxyterephthaldehyde (DMTP) as COF ligands and then decorated with AuNPs via the citrate reducing method. Modification of COF-AuNPs by the aptamer against *S. typhimurium* formed aptamer-COF-AuNPs (apt-COF-AuNPs) to selectively identify the target. If the samples contain *S. typhimurium*, the apt-COF-AuNPs attach to the target surface, leading to shielding of their catalytical sites and reduction in peroxidase-like activity. *S. typhimurium*-inhibited peroxidase activity of COF-AuNPs slowed down the oxidation of 3,3′,5,5′ -Tetramethylbenzidine (TMB) with H_2_O_2_, giving the samples a lighter blue color at set times. Consequently, the color variations from dark blue to light blue were positively correlated with an increase in bacteria concentration. Furthermore, to meet the needs of the on-site test, we developed a novel smartphone application (APP) to read the semi-quantitative result. This method has good accuracy and sensitivity, interesting specificity and stability, and is significant in food safety.

## Results

### Characterization of COFs, COF-AuNPs, and apt-COF-AuNPs

**Fig. 2:**
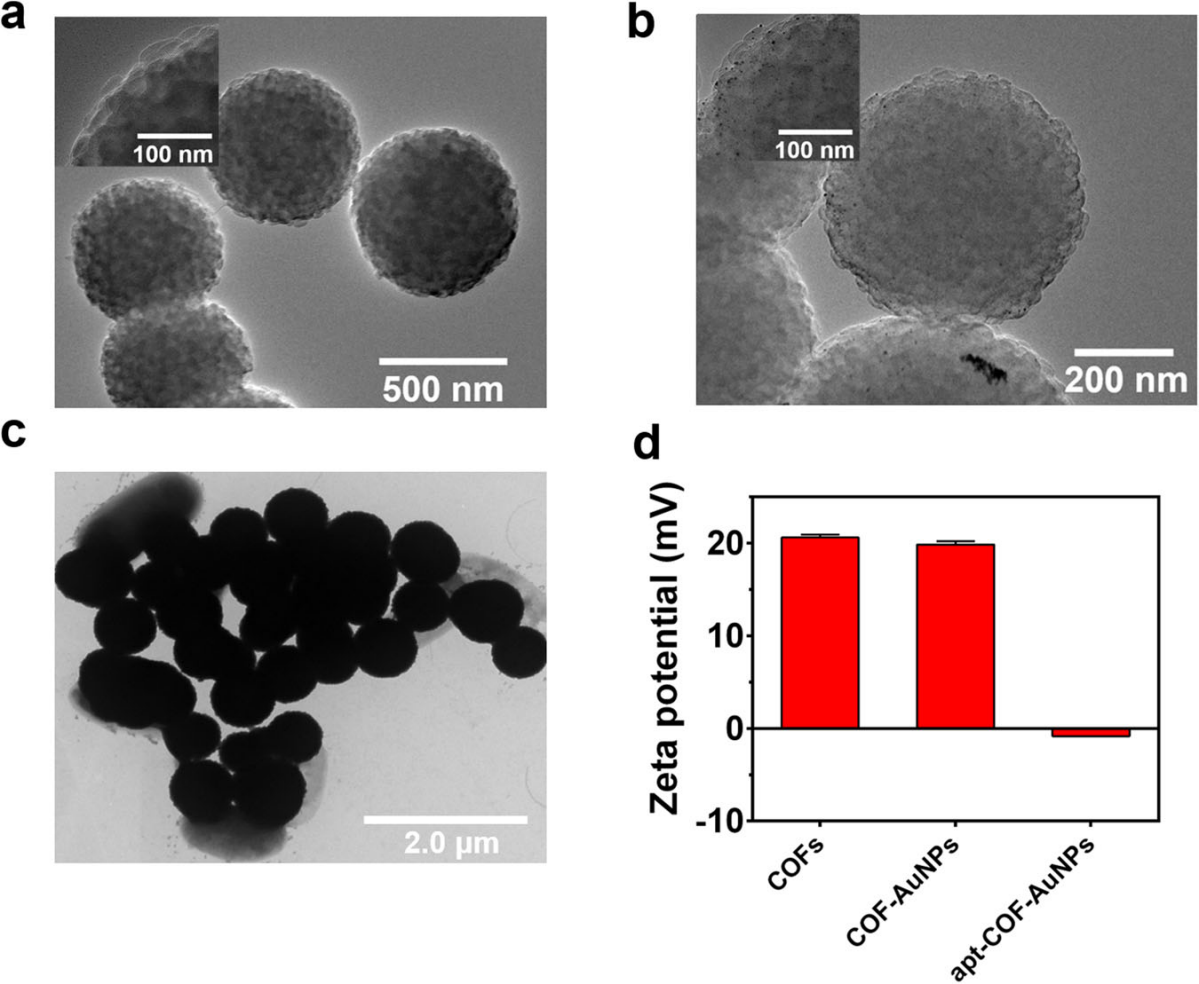
TEM images and Zeta potential. **a** COFs and **b** COF-AuNPs, **c** apt-COF-AuNPs combined with *S. typhimurium*. **d** Zeta potential values of COF, COF-AuNPs, apt-COF-AuNPs. In all samples, the final concentration of apt-COF-AuNPs is 0.2 mg/mL. The final concentration of *S. typhimurium* is 1 × 10^7^ CFU/mL. Error bars represent the standard deviation of three replicates.

The morphology characters of COFs and COF-AuNPs were recorded by transmission electron microscopy (TEM). Figure 2a, b both presented uniform spherical morphology of COFs and COF-AuNPs with a diameter of about 393.54 ± 27.34 nm. In Fig. 2b, the AuNPs were uniformly scattered on the COFs because of the coordination of the unsaturated amino groups of the COFs. In addition, the TEM photograph of apt-COF-AuNPs and *S. typhimurium* mixtures verified their tight-binding (Fig. 2c). To investigate the crystallinity of COF-AuNPs, the X-ray diffraction (XRD) patterns were demonstrated from 2*θ* = 5° to 80°. As shown in Supplementary Fig. 1, the result showed that the peaks at 38.22°, 44.22°, and 64.72° were well in accord with the reflections from (111), (200), and (220) planes of Au^32^. Energy-dispersive X-ray spectroscopy (EDS) elemental mapping results showed the presence of C, O, N, and Au on the Au-COF surface (Supplementary Fig. 2). The results demonstrated that AuNPs were successfully doped on COFs. Then, FT-IR characterizations were employed in verifying the modifying of aptamer on the COF-AuNPs (Supplementary Fig. 3). The obvious characteristic bands at 1411, 1643 and 3603 cm^−1^ can be ascribed to the stretching vibration of the C=N and N–H bond, indicating the successful synthesis of apt-COF-AuNPs. Moreover, after modification of aptamers on COF-AuNPs, the zeta potential displayed a decreased value from 19.83 to −0.84 mV (Fig. 2d) due to the negative charges taken by aptamers^33^. The results indicated that the apt-COF-AuNPs were successfully prepared.

### Feasibility verification

**Fig. 3:**
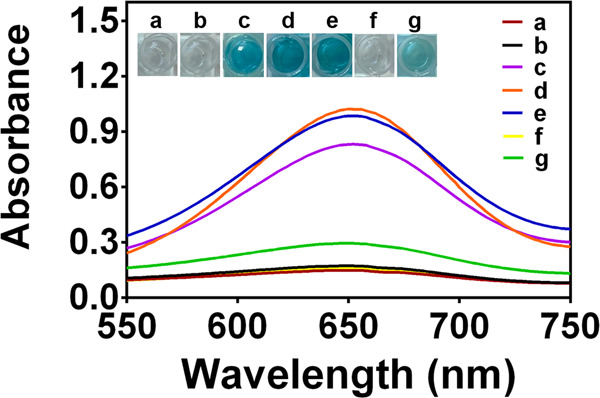
UV–Vis absorption spectra and photographs of different reaction systems. TMB + H_2_O_2_ (**a**), COF + TMB + H_2_O_2_ (**b**), AuNPs + TMB + H_2_O_2_ (**c**), COF-AuNPs + TMB + H_2_O_2_ (**d**), apt-COF-AuNPs + TMB + H_2_O_2_ (**e**), apt-COF-AuNPs + TMB (**f**), apt-COF-AuNPs + *S. typhimurium* + TMB + H_2_O_2_ (**g**). Reaction conditions were COF (0.2 mg/mL), COF-AuNPs (0.2 mg/mL), and apt-COF-AuNPs (0.2 mg/mL), *S. typhimurium* (1 × 10^7^ CFU/mL), TMB (2 mM) and H_2_O_2_ (2 M) at 37 °C.

The detection system was verified by the TMB-H_2_O_2_ system. Considering the absorption peak of the aggregated AuNPs at 650 nm may overlap with that of oxTMB product to affect the test results^34^, we used COF as the skeleton of AuNPs to avoid the color of probe itself affecting the colorimetric results. As shown in Supplementary Fig. 4, the COF-AuNPs have an extremely low absorption between 650 and 700 nm, therefore probe itself was almost no signal interference to the TMB-H_2_O_2_ system. As shown in Fig. 3, the COF cannot catalyze the TMB-H_2_O_2_ system to produce blue oxTMB, indicating that COF had no peroxidase activity (curve b). In contrast, AuNPs, COF-AuNPs, and apt-COF-AuNPs can catalyze the oxidation of TMB by H_2_O_2_ with an absorption peak at 652 nm (curves c, d, and e), which is similar to HRP (Eq. (1))^35^.

1 $${{{\mathrm{H}}}}_2{{{\mathrm{O}}}}_2 + {{{\mathrm{TMB}}}}^{\underrightarrow {{{{\mathrm{apt}}}} - {{{\mathrm{COF}}}} - {{{\mathrm{AuNPs}}}}}}{{{\mathrm{H}}}}_2{{{\mathrm{O}}}} + {{{\mathrm{oxTMB}}}}$$

Besides, no absorption peak appeared when apt-COF-AuNPs catalyzed TMB alone (curve f). This verified that apt-COF-AuNPs had no oxidase activity. Herein, COF-AuNPs (curve d) have better peroxide-mimetic enzyme activity than bare AuNPs (curve c), because the spherical COF can be the supporting frame of AuNPs to offer more enough reaction sites and space for catalyzing the TMB-H_2_O_2_ system^36^. And the excellent adsorption capacity of COF could improve the surrounding substrate concentration to further enhance the catalytic activity. When *S. typhimurium* was captured by apt-COF-AuNPs, the absorbance at 652 nm decreased, which demonstrated that *S. typhimurium* could mask the peroxidase-like activity of apt-COF-AuNPs (curve g). According to the above results, apt-COF-AuNPs can be used as a colorimetric biosensor for detecting *S. typhimurium* based on its peroxidase-like activity and *S. typhimurium*-induced shielding effect.

### Kinetic analysis of apt-COF-AuNPs

**Fig. 4:**
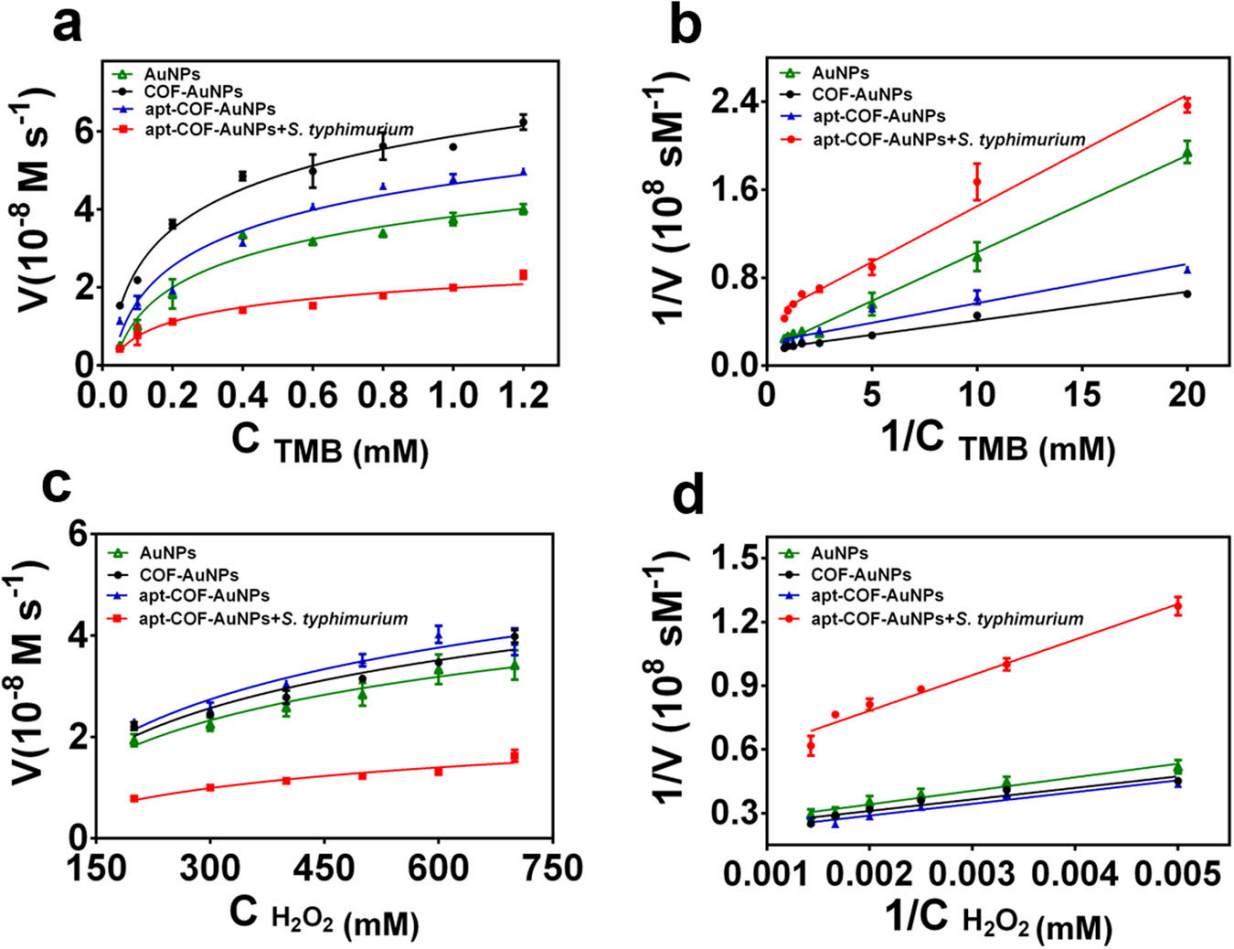
Catalytic kinetics analysis. **a** Steady-state kinetic assay and **b** double reciprocal plots of activity of AuNPs, COF-AuNPs, apt-COF-AuNPs, and apt-COF-AuNPs + *S. typhimurium*. The concentration of H_2_O_2_ was 2 M and the TMB concentration was varied. **c** Steady state kinetic assay and **d** double reciprocal plots of activity of AuNPs, COF-AuNPs, apt-COF-AuNPs, and apt-COF-AuNPs + *S. typhimurium*. The concentration of TMB was 2 mM and the H_2_O_2_ concentration was varied. Error bars represent the standard deviation of three replicates.

The kinetics constants maximal reaction velocity (*v*_max_) and Michaelis constant (*K*_*m*_) were obtained using different concentrations of the substrates TMB and H_2_O_2_. Typical Michaelis–Menten curves and the corresponding double reciprocal plots of AuNPs, COF-AuNPs, apt-COF-AuNPs, and apt-COF-AuNPs + *S. typhimurium* are shown in Fig. 4. *K*_*m*_ represents the affinity of the enzyme for its substrate, and *K*_*m*_ value is lower, the enzymes affinity to substrates is higher^35^. The *K*_*m*_ of AuNPs, COF-AuNPs, and apt-COF-AuNPs towards TMB were similar but lower than apt-COF-AuNPs-*S. typhimurium* composite (Supplementary Table 1). The same result could be obtained when H_2_O_2_ is the substrate. The affinity to substrate and *v*_max_ of COF-AuNPs was superior to bare AuNPs. These results also revealed that the affinity of COF-AuNPs to substrate hardly changed after modification with aptamers, but decreased after binding to the *S. typhimurium*. In addition, COF-AuNPs and apt-COF-AuNPs had higher *v*_max_ for TMB or H_2_O_2_, which verified that apt-COF-AuNPs-*S. typhimurium* composite needed a higher substrate concentration to reach the maximum activity.

In addition, the stability of AuNPs and apt-COF-AuNPs was evaluated. As shown in Supplementary Fig. 5a, the peroxidase-like activity of apt-COF-AuNPs kept stable at pH 2–6. When the buffer pH further increased in the pH range of 8–12, a drop in the absorbance was observed. This drop should be due to the instability of H_2_O_2_ in alkaline solutions^37^. However, the peroxidase-like activity of AuNPs only kept stable at pH 4–6. According to a previous study, the AuNPs can aggregate in pH < 4 solutions^38^, resulting in the inhibition of catalytic activity^39^. Therefore, modification of AuNPs on the spherical COF improved pH stability, especially in acidic conditions. Furthermore, in the temperature range of 4–80 °C (Supplementary Fig. 5b), apt-COF-AuNPs can still maintain a better catalytic effect than AuNPs, and compared with the natural enzyme has great advantages. Then, we explored the storage lifetime of the apt-COF-AuNPs (Supplementary Fig. 6). The statistical analysis (one-way ANOVA) showed no statistical difference in the catalytic activity of freshly synthesized nanocomposite and the one stored for 1 to 6 months (*F* = 0.520, *p* > 0.05). This prepared apt-COF-AuNPs was stable for at least 6 months. The above results indicated that the apt-COF-AuNPs had good stability.

### Optimization of experimental conditions

The experimental conditions of this system were optimized to obtain the best assay performance. All experiment details and results were shown in the Supplementary Information (Supplementary Fig. 7). The experimental conditions are selected as follows: (a) incubation time: 30 min. (b) Coloration time: 5 min.

### Sensitivity and selectivity of the aptasensor

**Fig. 5:**
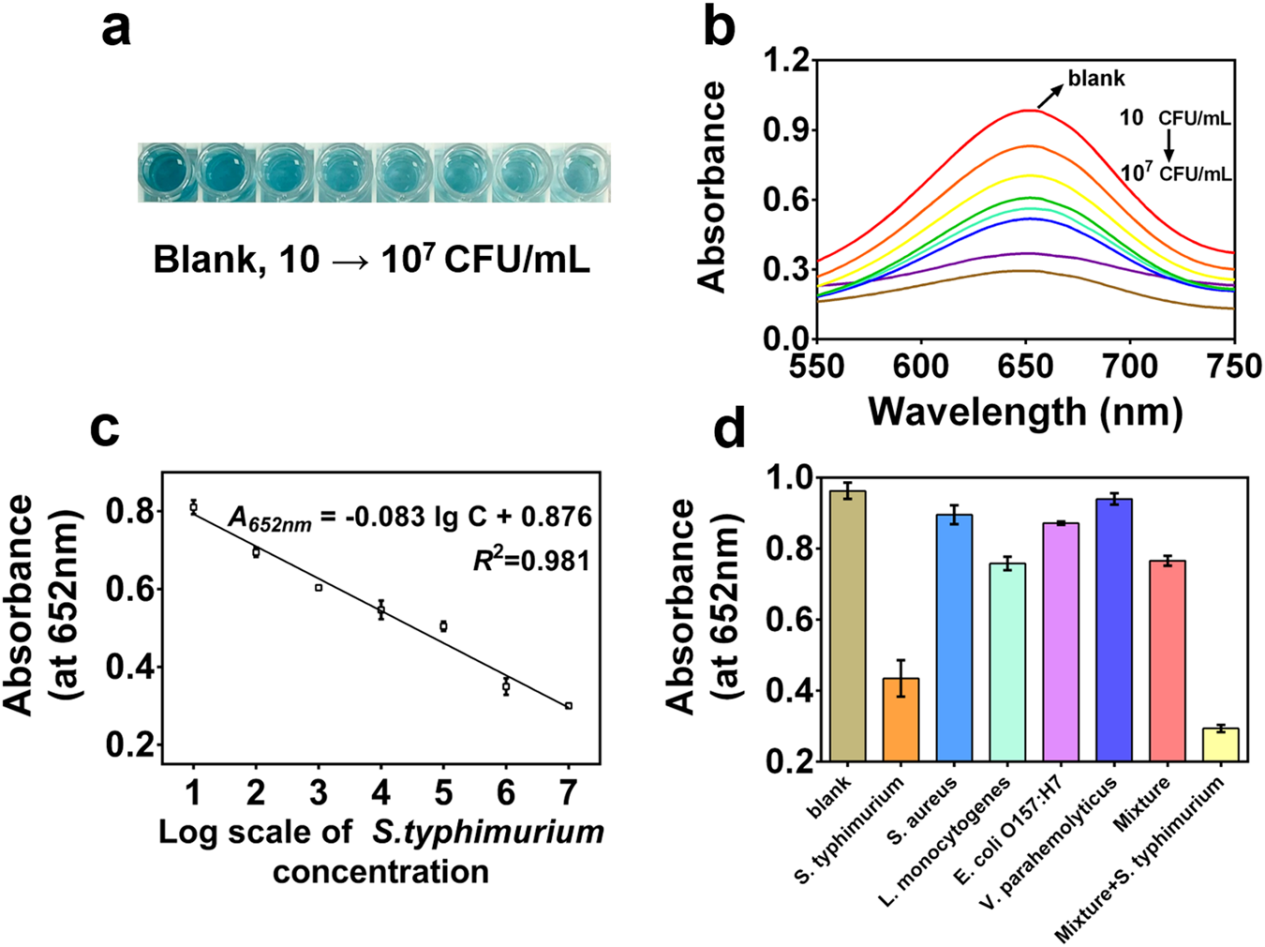
Sensitivity and selectivity. **a** Photographs and **b** UV–Vis spectra of the colorimetric assay with different concentrations of *S. typhimurium* (from 1 × 10 to 1 × 10^7^ CFU/mL), **c** the calibration curve for detecting *S. typhimurium* (the *A*_*652nm*_ value vs. the log scale of *S. typhimurium* concentration), **d** the *A*_*652nm*_ value of colorimetric results of different bacteria samples (blank, *S. typhimurium*, *S. aureus*, *L. monocytogenes*, *E. coli O157:H7*, *V. parahaemolyticus*, mixture, and mixture + *S. typhimurium*). Error bars represent the standard deviation of three replicates.

Under the optimal experimental conditions, various concentrations of *S. typhimurium* were detected using this aptasensor. In Fig. 5a, the blue color of the solution gradually deepened as the bacterial concentration increased, corresponding to the change in UV–Vis spectra (Fig. 5b). The absorbance at 652 nm (*A*_652 nm_) increased with the *S. typhimurium* concentration from 1 × 10 to 1 × 10^7^ CFU/mL, and *A*_652 nm_ showed a well linear relationship with the logarithm of *S. typhimurium* concentration (C): $A_{652\;{{{\mathrm{nm}}}}} = 0.083\lg C + 0.876,\;R^2 = 0.981$, where *R*^2^ is the correlation coefficient (Fig. 5c). The limit of detection (LOD) of this aptasensor was 7 CFU/mL (LOD = 3SD/slope, where SD is the standard deviation of blank samples and the slope is obtained from the standard curve)^16^.

**Fig. 6:**
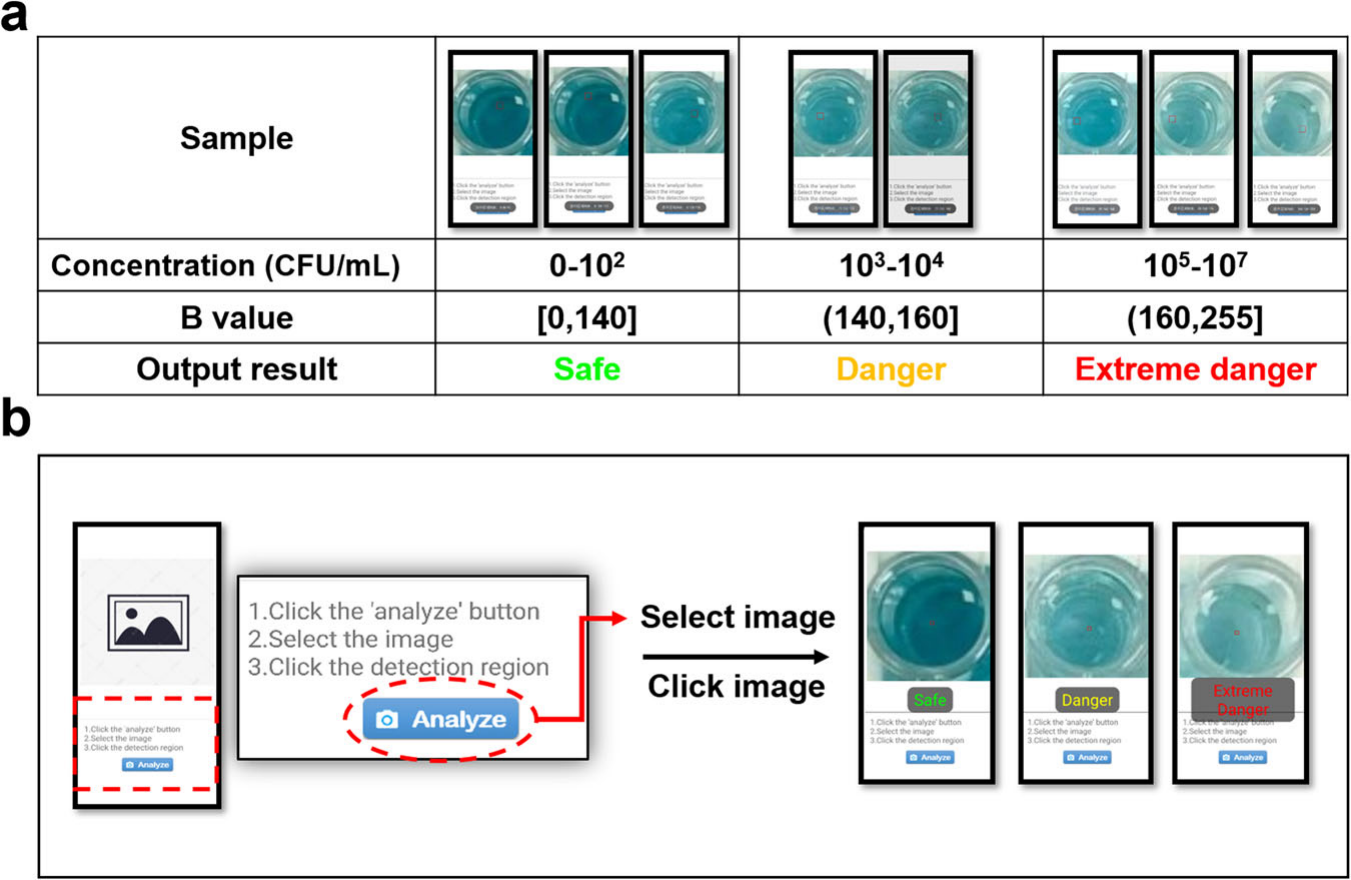
Proof-of-concept smartphone APP for analysis of *S. typhimurium*. **a** Calibration of the smartphone APP by using a standard image, **b** semi-quantitative analysis of *S. typhimurium* using a proof-of-concept smartphone APP. All images created by authors.

Then, we constructed a proof-of-concept smartphone APP for the semi-quantitative analysis of *S. typhimurium*. And the APP was calibrated by a series of standard images (Fig. 6a). The standard images were obtained by assaying *S. typhimurium* with concentrations from 1 × 10 to 1 × 10^7^ CFU/mL. As shown in Supplementary Fig. 8, the *B* value showed a linear relationship with the logarithm of *S. typhimurium* concentration (C): ${{{{B}}}}\;{{{\mathrm{value}}}} = 11.43\lg C + 107.20,\;R^2 = 0.947$, the LOD was 12 CFU/mL (LOD = 3SD/slope). The *S. typhimurium* concentration in the samples below 1 × 10^2^ CFU/mL is considered safe because the infectious dose for *S. typhimurium* is 1 × 10^3^ CFU/mL^40^. According to the range of B value, the risk level is output as the semi-quantitative analysis result (Fig. 6b). This APP is expected to be an efficient tool to assist field detection.

To study the selectivity of this method, we tested 1 × 10^6^ CFU/mL of *S. typhimurium*, 1 × 10^7^ CFU/mL of four other common bacterial strains (*Staphylococcus aureus* (*S. aureus*, ATCC 23213), *Listeria monocytogenes* (*L. monocytogenes*, ATCC 43251), *Escherichia coli O157:H7* (*E. coli O157:H7*, ATCC 43888), and *Vibrio parahaemolyticus* (*V. parahaemolyticus*, ATCC 17802)), the mixture of four other common bacterial strains, and the mixture with *S. typhimurium*. As shown in Fig. 5d, the absorbance at 652 nm decreased remarkably when *S. typhimurium* was presented in samples. The result proved that this method is highly selective for *S. typhimurium* on account of the specific affinity between the aptamers and the outer membrane proteins and lipopolysaccharides of *S. typhimurium*^41^.

The above results indicated that this method has excellent sensitivity and selectivity. As shown in Table 1, compared with previously reported colorimetric methods, we have obvious advantages in detection time and detection LOD. In addition, our method is comparable to other methods such as electrochemical, fluorescence, and SERS methods in detection effect, which is less dependent on large equipment platform and easier to operate. Additionally, this method doesn’t need pre-enrichment and the entire testing process takes only 40 min, including the incubation of apt-COF-AuNPs with sample and centrifugal separation (35 min), and the reaction time of apt-COF-AuNPs + *S. typhimurium* and TMB-H_2_O_2_ system (5 min). Therefore, this method also has an obvious advantage in detection time.

**Table 1:** A brief overview of recently reported methods for the determination of *S. typhimurium*.

Materials used	Detection method	Linear range (CFU/mL)	LOD (CFU/mL)	Detection time	Reference
Gold/silver nanodimer	SERS	10^2^–10^7^	50	45 min	^9^
Alendronic acid @ upconversion nanoparticles and AuNPs	Fluorescence	1.16 × 10^2^–1.16 × 10^7^	36	21 min	^8^
Antibody modified nickel nanowires	Electrochemical	10^2^–10^6^	80	2 h	^42^
AuNPsmagnetic particles	Colorimetric	10^3^–10^8^	1000	3 h	^43^
Aptamers@BSA-AuNC	Colorimetric	10^1^–10^6^	1	>1 h	^44^
Aptamer-functionalized magnetic beads and AuNPs	Colorimetric	10^2^–10^6^	240	>3 h	^45^
apt-COF-AuNPs	Colorimetric	10^1^–10^7^	7	40 min	This work

### Analysis of real samples

The applicability of this method was evaluated by assay of the lake water (from Chaoyang Lake and Nanhu Lake) and milk (from Mengniu Dairy) samples contaminated by *S. typhimurium*. The milk samples were spiked with *S. typhimurium* at various concentrations for establishing the calibration curve to eliminate matrix interferences (Supplementary Fig. 9). The lake water and milk contaminated by *S. typhimurium* were analyzed in parallel by plate counting method (Supplementary Fig. 10) and our method. The recovery rates are 109.37, 101.20, and 102.72% by testing *S. typhimurium* in milk and the water from Nanhu Lake and Chaoyang Lake (Table 2). And the relative standard derivations (RSDs) were all below 10%, which suggested that this aptasensor can be reliable and useful for analyzing *S. typhimurium* in real samples.

**Table 2:** Recovery and RSD values of detecting *S. typhimurium* in real samples $\left( {{\it{\bar x}} \pm {\it{s}},\;{{{n}}} = 3} \right)$.

Sample	Plate count result (log C, CFU/mL)	Recovered (log C, CFU/mL)	Recovery (%)	RSD (%)
Milk	3.46 ± 0.02	3.78 ± 0.27	109.37	7.23
Water from Nanhu Lake	2.82 ± 0.01	2.89 ± 0.08	101.20	2.60
Water from Chaoyang Lake	3.45 ± 0.01	3.49 ± 0.09	102.72	2.60

## Discussion

In this study, we constructed a simple, and rapid optical sensor based on target-induced masking of the peroxidase-like activity of apt-COF-AuNPs for *S. typhimurium* detection. The apt-COF-AuNPs are easy to synthesize and have high peroxidase-like activity and stability. Compared to current colorimetric methods, this method was simple and sensitive. In addition, combined with the developed smartphone APP, the results could be semi-quantitative graded, providing a powerful tool for on-site detection. It is envisaged that our colorimetric aptasensor would be widely applied for the fast detection of other pathogens using the respective aptamers. In conclusion, this method has many key bioanalytical properties including high sensitivity and selectivity, short time, easy operation, and low cost, making it an efficient method for the determination of *S. typhimurium* in food and environmental samples.

## Methods

### Materials and reagents

TAPB, DMTP, chloroauric acid (HAuCl_4_⋅3H_2_O, 99%), and TMB were from Macklin Biochemical (China). The thiol modified aptamer of *S. typhimurium* was synthesized by Sangon Biotech Co., Ltd. (China). This ssDNA aptamer has been confirmed sequence-specific binding to *S. typhimurium* outer membrane protein^41^. The sequence is as follows: 5’-SH-(CH_2_)6-TAT GGC GGC GTC ACC CGA CGG GGA CTT GAC ATT ATG ACA G-3’. Reagents such as H_2_O_2_ were all obtained from Beijing Chemical Reagent (China). Phosphate buffer solution (PBS, 0.01 M, pH 7.4) was from Sangon Biotech Co., Ltd. (China).

### Instruments

The UV–Vis absorption spectra were obtained by a spectrophotometer (TU-1810 DPC Persee, China). TEM images were performed on a JEOL JEM-2100F transmission electron microscope (JEOL, Japan). Fourier transform infrared (FTIR) spectra were recorded as KBr disks on a Nicolet 6700 FTIR spectrometer (Thermo Fisher Scientific, USA). X-ray diffraction (XRD) patterns were recorded on a D2 PHASER diffractometer (Bruker, German).

### Bacterial culture

Five foodborne pathogenic bacterial strains ((*S. typhimurium* (ATCC 14028) *Staphylococcus aureus* (*S. aureus*, ATCC 23213), *Listeria monocytogenes* (*L. monocytogenes*, ATCC 43251), *Escherichia coli O157:H7* (*E. coli O157:H7*, ATCC 43888), and *Vibrio parahaemolyticus* (*V. parahaemolyticus*, ATCC 17802)) were from the Department of Hygienic Inspection, School of Public Health, Jilin University, China. All of them were grown on Luria-Bertani agar plates, except *V. parahaemolyticus*, which was on LB plates with 3% (*w/v*) NaCl. After 24 h of inoculation, a single colony of each strain was picked up and incubated in corresponding Luria-Bertani medium at 37 °C with a shaking at 180 rpm for 12 h. Then, the bacteria were inactivated by the 24 h-incubation of 4% formaldehyde at 4 °C. Lastly, the bacteria were washed three times using sterile PBS by centrifugation at 4000 rpm for 15 min. 100 μL of the diluted suspensions was plated onto appropriate agar plates by spread plating. Bacterial counts were performed on plates, and the total bacterial counts (CFU per mL) were log transformed. The logarithm of quantitative concentration bacterial fluid was used to evaluate the effect of our assay method.

### Synthesis of apt-COF-AuNPs

The COFs were manufactured based on a previously reported method^36^. Briefly, 105.4 mg of TAPB (0.3 mmol) and 87.4 mg of DMTP (0.45 mmol) were ultrasonically dissolved in the blend solution (20 mL of 1,4-dioxane, 20 mL of butanol, and 20 mL of methanol). Then, 500 μL of 12 M aqueous acetic acid was added to the homogeneous solution and the mixed solution was placed at 25 °C for 2 h. Afterward, 4.5 mL of 12 M aqueous acetic acid was added again, and the mixture reacted at 70 °C for 24 h. Lastly, the TAPB-DMTP-COFs were washed with tetrahydrofuran twice and acetone twice. The collected COFs were dried in a vacuum overnight.

60 mg of COFs and 320 μL HAuCl_4_ (1%) were added into 30 mL of methanol solution (pH = 4.0) and stirred at 0 °C for 5 h. 2 mL NaBH_4_ methanol solution (0.20 M) was added to the mixture and the reaction was continued at 0 °C for 3 h. Subsequently, the resulting precipitate was washed three times with methanol. Finally, the obtained COF-AuNPs were dried vacuum oven for 24 h. The bare AuNPs were prepared in the same way as COF-AuNPs without COFs. In briefly, 320 μL HAuCl_4_ (1%) were added into 30 mL of DDW and stirred at 0 °C for 10 min. 2 mL NaBH_4_ solution (0.20 M) was added to the mixture and the reaction was continued at 0 °C for 1 h.

2 mg of COF-AuNPs was dissolved in 1 mL DDW by ultrasonication to get a homogeneous solution. 10 μL of aptamers (10 μM) were heated at 92 °C for 5 min and added in 2 mg/mL COF-AuNPs, with a slight stirring overnight for the combination of COF-AuNPs via Au–S bond. Finally, apt-COF-AuNPs were washed three times with DDW for further use.

### Investigation of the peroxidase-like activity of COFs, AuNPs, COF-AuNPs, and apt-COF-AuNPs

To investigate the peroxidase mimic activity of material (COFs, AuNPs, COF-AuNPs, and apt-COF-AuNPs). Firstly, 100 μL of 0.2 mg/mL material was added to 900 μL mixture containing 0.2 M acetid acid (HAc)-sodium acetate (NaAc) buffer (pH = 4.0), 2 mM TMB and 2 M H_2_O_2_. Then, the absorbance at 652 nm of samples was measured by a UV–Vis spectrophotometer. The peroxidase activity assays can be carried out using the initial reaction rate, which was calculated by the molar absorption coefficient of colorimetric substrate TMB (*ε*_652 nm_ = 39,000/(M × cm)^35^. The effect of TMB or H_2_O_2_ concentration on the reaction velocity changes was studied to get the kinetic parameters by the Michaelis-Menten equation (Eq. (2))^35^.

2 $$v = \frac{{v_{{\rm{max}}} \times \left[ {{{\mathrm{S}}}} \right]}}{{K_m + \left[ S \right]}}$$

In Eq. (2), *v* is the initial reaction velocity, *v*_max_ is the maximal reaction velocity, [*S*] is the substrate concentration and *K*_*m*_ is the Michaelis constant^35^.

#### Optimization of experimental conditions

The main parameters of the assay procedure were performed to enhance the assay sensitivity and reliability and minimize the total analysis time, including the incubation time of apt-COF-AuNPs and *S. typhimurium*, reaction temperature, pH and reaction time of apt-COF-AuNPs and TMB-H_2_O_2_ system. First, different incubation time (15, 30, 45, 60, 75 min) were used to detect positive samples (1 × 10^7^ CFU/mL *S. typhimurium*). It can be seen from Supplementary Fig. 7a that the value of absorbance at 652 nm was decreased to a plateau in 30 min. Then, a series of different coloration time (2, 3, 4, 5, 6, 7, 8 min) for TMB-H_2_O_2_ system was used to detect positive samples (1 × 10^7^ CFU/mL *S. typhimurium*) and negative samples (staired PBS). It can be seen from Supplementary Fig. 7b that the value of absorbance at 652 nm between negative and positive was largest at 5 min. Based on the above results, the following experimental conditions were chosen to obtain the best assay results: (a) incubation time: 30 min. (b) coloration time: 5 min.

### Procedures for *S. typhimurium* detection

The stock solution of *S. typhimurium* was diluted by sterile PBS to the standard solutions of different concentrations (1 × 10–1 × 10^7^ CFU/mL). Under the optimized experimental conditions, 900 μL of the standard solution was added to 100 μL of 2 mg/mL apt-COF-AuNPs with 30 min stirring. Next, the mixtures were centrifuged at 4000 rpm for 5 min and were removed supernatant. Then, the HAc-NaAc buffer containing 2 M H_2_O_2_ and 2 mM TMB was added to the above precipitate with a reaction of 10 min at 37 °C. Last, the UV absorption spectral and absorbance at 652 nm were measured by a UV–Vis spectrophotometer. To test assay results in lake water (from Chaoyang Lake and Nanhu Lake) and milk (from Mengniu Dairy) samples, the detection procedure was described above, except for replacing the PBS buffer with lake water and milk.

### The development and validation proof-of-concept smartphone APP

To achieve field testing, a novel proof-of-concept smartphone APP (Android system) was developed. This APP has the function of image acquisition and analysis based on the three primary colors: red, green, and blue (RGB) model. Smartphone cameras use complementary metal-oxide-semiconductor (CMOS) imaging sensors that only detect the wavelengths of RGB. Working directly with the unaltered as-measured RGB values is generally preferable when applying smartphones to colorimetric readers. According to the color change of the H_2_O_2_-TMB system, the blue value (B value) showed a better quantitative correlation in this detection method. Based on the above, the APP was developed through the Android Studio integrated development environment by IT professionals. In order to complete the software development efficiently and reasonably, we assayed needs and designed the software use process, and then divided the software into three relatively independent sub-modules according to the requirements and processes namely: image acquisition module, data analysis and display module, and data storage module. The image acquisition module is for capturing images of samples. The data analysis and display module are for calculating the B value and translating to the risk level (Safe: 0–10^2^ CFU/mL, Danger: 10^3^–10^4^ CFU/mL, Extreme danger: 10^5^–10^7^ CFU/mL). The data storage module is using for saving images and data. The software development steps are as follows: (1) start a new Android Studio project; (2) write logical program in mainactivity.java and control the layout in activity_main.xml; (3) Simulate by simulator; (4) run APP.

The operation steps of the APP were simple: (1) Click the “analyze” button on the home page; (2) select the obtained photograph of the testing result; (3) click the detection region. The codes of the APP were in Supplementary Information.

## Supplementary information

Supplementary information

## Data Availability

The data supporting the finding reported herein are available on reasonable request from the corresponding author.

## Code availability

Code for data analysis is available in Supplementary Information.
