# Supplementary material for: On-site colorimetric detection of Salmonella typhimurium
Source: NPJ Sci Food. 2022 Oct 17;6:48. doi: 10.1038/s41538-022-00164-0 (PMC9576709; doi:10.1038/s41538-022-00164-0)
Supplement: Supplementary file 1 — Supplementary information [file 41538_2022_164_MOESM1_ESM.pdf]

On-site colorimetric detection of *Salmonella typhimurium*

Shengnan Wei, Zhenyue Su, Xiangong Bu, Xuening Shi, Bo Pang, Liang Zhang, Juan Li*, Chao Zhao*

School of Public Health, Jilin University, Changchun, Jilin, 130021, China

* E-mail: li_juan@jlu.edu.cn; czhao0529@jlu.edu.cn

CONTENT

1. Supplementary Figures and Tables

Supplementary Figure. 1 The characterization of COF-AuNPs (XRD pattern).

Supplementary Figure. 2 The characterization of COF-AuNPs (EDS elemental mapping analysis).

Supplementary Figure. 3 The characterization of COF, COF-AuNPs, and apt-COF-AuNPs (FT-IR spectra).

Supplementary Figure. 4 The signal interference verification of apt-COF-AuNPs.

Supplementary Figure. 5 PH and temperature stability.

Supplementary Figure. 6 Storage stability.

Supplementary Figure. 7 The optimization results of several parameters.

Supplementary Figure. 8 The calibration curve acquired by the proof-of-concept smartphone APP.

Supplementary Figure. 9 The calibration curve acquired by spiked milk samples.

Supplementary Figure. 10 The plating culture results of *S. typhimurium* in real samples.

Supplementary Table 1 Comparison of K_m and v_{max} of COF-AuNPs, apt-COF-AuNPs and apt-COF-AuNPs + S. *typhimurium* for TMB and H_2O_2.

2. Codes of the APP on Smartphone

1. Supplementary Figures and Tables.

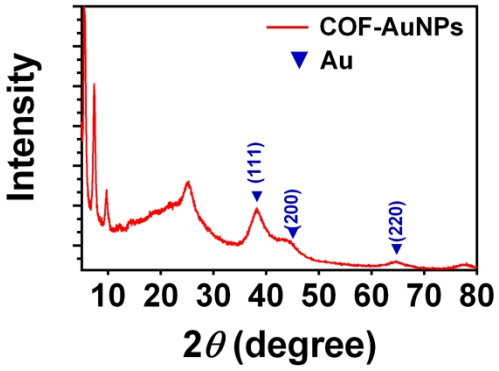

Supplementary Figure. 1 The characterization of COF-AuNPs (XRD pattern). Wide-angle XRD pattern of COF-AuNPs.

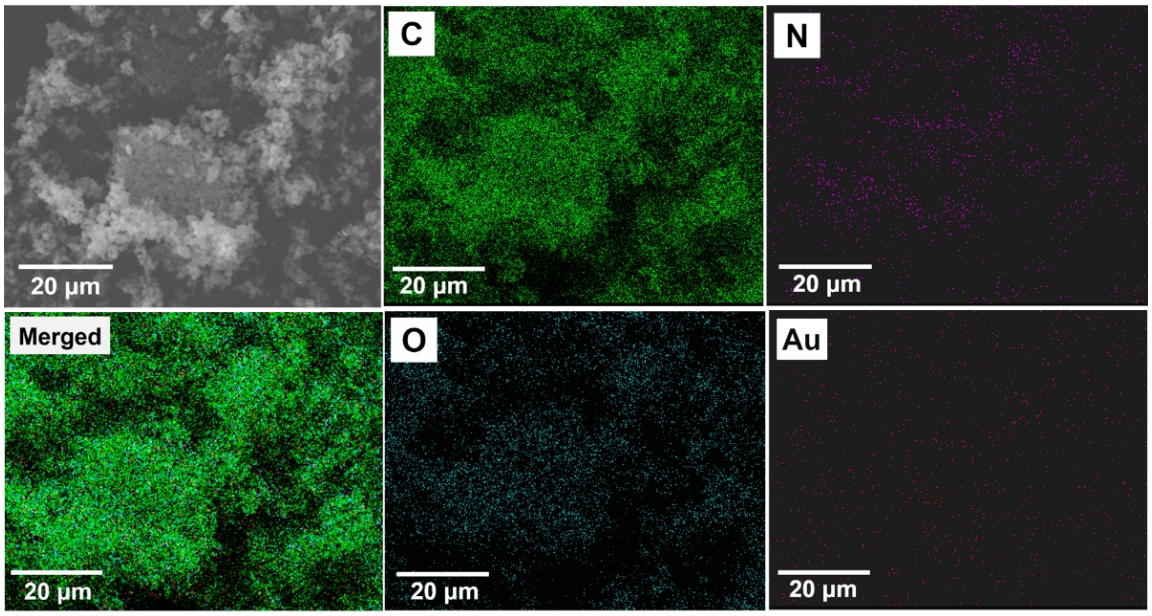

Supplementary Figure. 2 The characterization of COF-AuNPs (EDS elemental mapping analysis). EDS elemental mapping used in conjunction with dark field scanning-TEM taken over the particle shows carbon (green), nitrogen (purple), oxygen (blue) and gold (red) present; the scale bars are 20 μm.

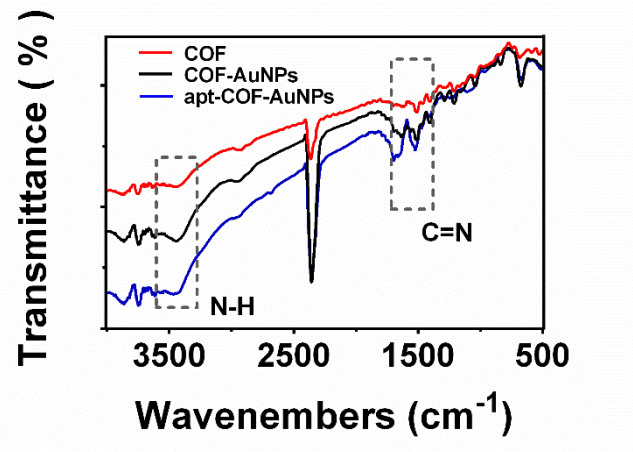

Supplementary Figure. 3 The characterization of COF, COF-AuNPs, and apt-COF-AuNPs (FT-IR spectra). FT-IR specra of COF (red line), COF-AuNPs (black line) and apt-COF-AuNPs (blue line).

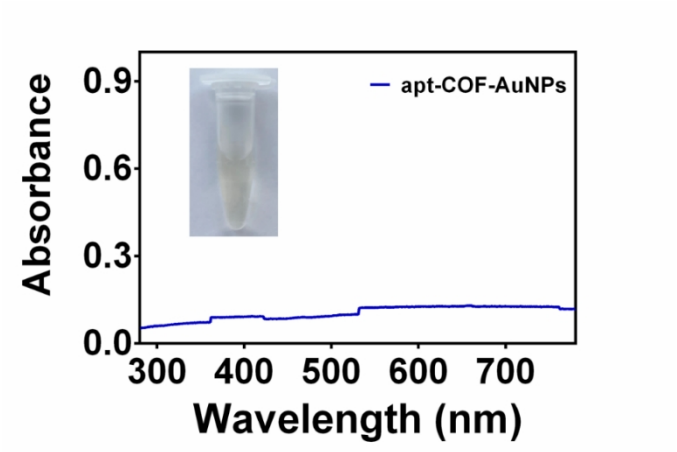

Supplementary Figure. 4 The signal interference verification of apt-COF-AuNPs. The photograph and uv-vis spectrum of apt-COF-AuNPs.

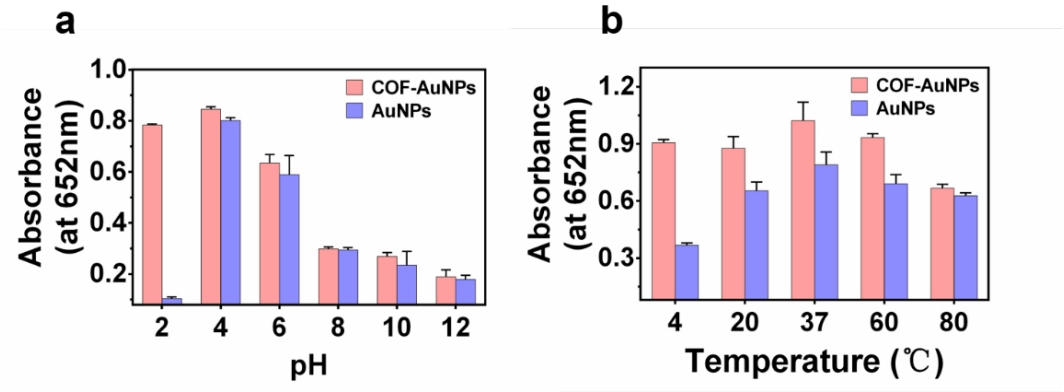

Supplementary Figure. 5 PH and temperature stability. UV-Vis absorption at 652 nm of TMB-H_2O_2 system catalyzed by apt-COF-AuNPs with different pH (at 37℃) and **b** different temperature (at pH = 4.0). Error bars represent the standard deviation of three replicates.

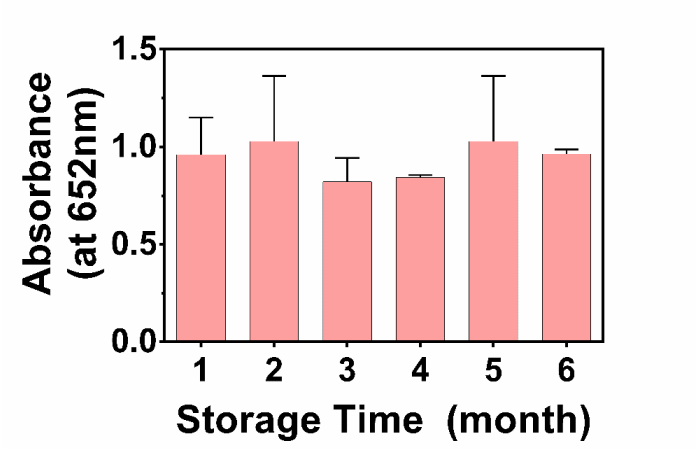

Supplementary Figure. 6 Storage stability. UV-Vis absorption at 652 nm of TMB-H_2O_2 system catalyzed by apt-COF-AuNPs with storage for one to six months. Error bars represent the standard deviation of three replicates.

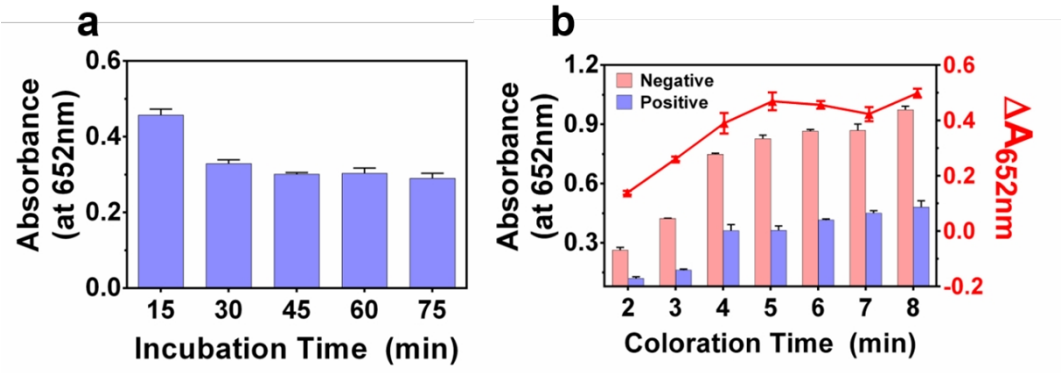

Supplementary Figure. 7 The optimization results of several parameters. a incubation time of apt-COF-AuNPs and *S. typhimurium*, **b** coloration time of TMB-H_2O_2 system catalyzed by apt-COF-AuNPs + *S. typhimurium* or apt-COF-AuNPs. Error bars represent the standard deviation of three replicates.

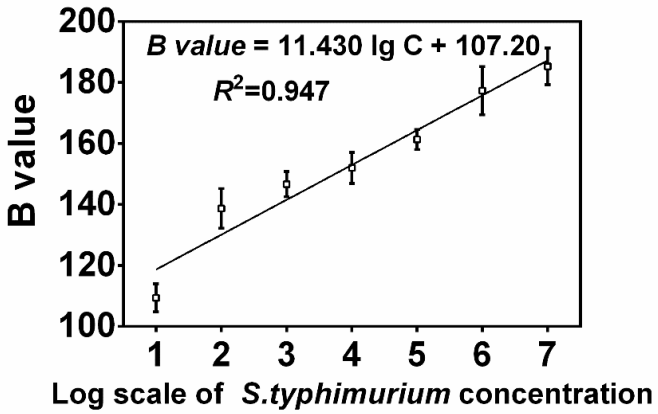

Supplementary Figure. 8 The calibration curve acquired by the proof-of-concept smartphone APP. The calibration curve for detecting *S. typhimurium* by the proof-of-concept smartphone APP. (*B value* vs. the log scale of *S. typhimurium* concentration). Error bars represent the standard deviation of three replicates.

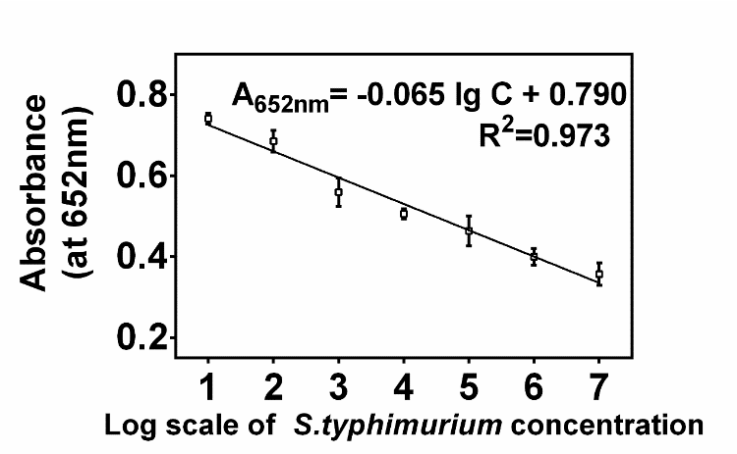

Supplementary Figure. 9 The calibration curve acquired by spiked milk samples. The calibration curve for detecting *S. typhimurium* in spiked milk samples (the A_{652nm} value vs. the log scale of *S. typhimurium* concentration). Error bars represent the standard deviation of three replicates.

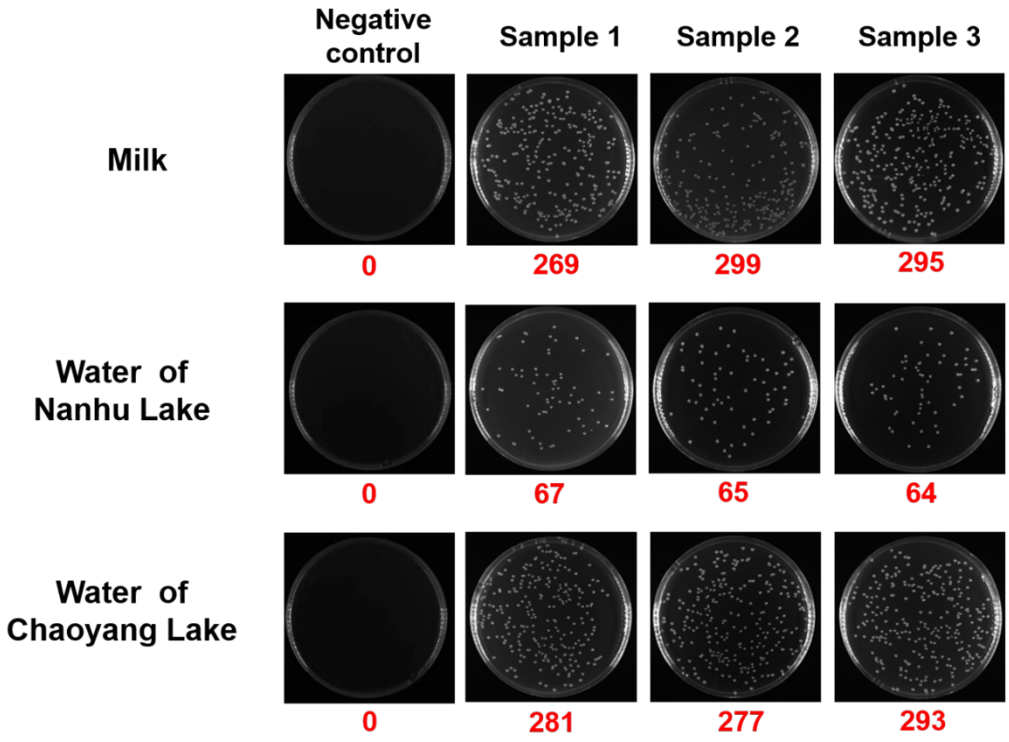

Supplementary Figure. 10 The plating culture results of *S. typhimurium* in real samples. Photographs of the colony-forming units on LB agar plates after treatment with real samples (milk, lake water of Nanhu Lake and Chaoyang Lake) for 12 h.

Supplementary Table 1. Comparison of K_m and v_{max} between AuNPs, COF-AuNPs, apt-COF-AuNPs and apt-COF-AuNPs + S. *typhimurium* for TMB and H_2O_2.

Catalyst	Substance	K_m (mM)	v_{max} ($M \cdot s^{-1}$)
AuNPs	TMB	0.5920	6.7114×10^{-8}
COF-AuNPs	TMB	0.1725	6.6094×10^{-8}
apt-COF-AuNPs	TMB	0.1680	4.7059×10^{-8}
apt-COF-AuNPs + S. *typhimurium*	TMB	0.2309	2.2815×10^{-8}
AuNPs	H_2O_2	298.3832	4.6729×10^{-8}
COF-AuNPs	H_2O_2	266.6667	4.9383×10^{-8}
apt-COF-AuNPs	H_2O_2	309.2894	5.5960×10^{-8}
apt-COF-AuNPs + S. *typhimurium*	H_2O_2	373.1430	2.2306×10^{-8}

2. Codes of the APP on Smartphone

```
1. package com.color.identify;
2. import android.content.Context;
3. import android.graphics.Bitmap;
4. import android.graphics.Canvas;
5. import android.graphics.Color;
6. import android.graphics.Paint;
7. import android.util.AttributeSet;
8. import android.util.Log;
9. import android.view.Gravity;
10. import android.view.LayoutInflater;
11. import android.view.MotionEvent;
12. import android.view.View;
13. import android.widget.ImageView;
14. import android.widget.TextView;
15. import android.widget.Toast;
16.
17. import androidx.annotation.Nullable;
18. import androidx.palette.graphics.Palette;
19.
20. public class RectImageView extends androidx.appcompat.widget.AppCompatImageView {
21.     private static final int WEIGHT = 100;
22.     private static final int HEIGHT = 100;
23.     int x = -1;
24.     int y;
25.     int xw;
26.     int yh;
27.
28.     private static final String TAG = "RectImageView";
29.
30.     public RectImageView(Context context) {
31.         super(context);
32.     }
33.
34.     public RectImageView(Context context, AttributeSet attrs) {
35.         super(context, attrs);
36.     }
37.
38.     public RectImageView(Context context, AttributeSet attrs, int defStyleAttr) {
39.         super(context, attrs, defStyleAttr);
40.     }
```

```
41.
42.     @Override
43.     protected void onDraw(Canvas canvas) {
44.         super.onDraw(canvas);
45.         Paint paint = new Paint();
46.         paint.setAntiAlias(true);
47.         paint.setColor(Color.RED);
48.         paint.setStrokeWidth(5);
49.         paint.setStyle(Paint.Style.STROKE);
50.         if (x != -1) {
51.             canvas.drawRect(x, y, xw, yh, paint);
52.         }
53.     }
54.
55.     @Override
56.     public boolean onTouchEvent(MotionEvent event) {
57.         //手指按下
58.         try {
59.             setDrawingCacheEnabled(true);
60.             Bitmap mPic = getDrawingCache();
61.             if (event.getAction() == MotionEvent.ACTION_DOWN &&
    mPic != null) {
62.                 Log.d(TAG, mPic.getWidth() + " " + mPic.getHeight() + " " +
    event.getX() + " " + event.getY());
63.                 if (event.getX() < mPic.getWidth() && event.getY() <
    mPic.getHeight()) {
64.                     x = (int) event.getX();
65.                     y = (int) event.getY();
66.                     xw = x + WEIGHT;
67.                     yh = y + HEIGHT;
68.                     Log.e(TAG, " " + x + " " + y + " " + xw + " " + yh);
69.                     Bitmap selectPic = mPic.createBitmap(mPic, x, y,
    WEIGHT, HEIGHT);
70.                     Palette palette = Palette.from(selectPic).generate();
71.                     Palette.Swatch vibrant = palette.getVibrantSwatch();
72.                     Palette.Swatch vibrant1 =
    palette.getDarkVibrantSwatch();
73.                     Palette.Swatch vibrant2 = palette.getMutedSwatch();
74.                     Palette.Swatch vibrant3 =
    palette.getLightVibrantSwatch();
75.                     Palette.Swatch vibrant4 = palette.getDarkMutedSwatch();
76.                     Palette.Swatch vibrant5 = palette.getLightMutedSwatch();
77.                     int rgb1 = -2;
78.                     if (vibrant != null) {
```

```
79.                                 rgb1 = vibrant.getRgb();
80.                             } else if (vibrant1 != null) {
81.                                 rgb1 = vibrant1.getRgb();
82.                             } else if (vibrant2 != null) {
83.                                 rgb1 = vibrant2.getRgb();
84.                             } else if (vibrant3 != null) {
85.                                 rgb1 = vibrant3.getRgb();
86.                             } else if (vibrant4 != null) {
87.                                 rgb1 = vibrant4.getRgb();
88.                             } else if (vibrant5 != null) {
89.                                 rgb1 = vibrant5.getRgb();
90.                             }
91.
92.                             Log.e("RectImageView ", "onTouch: rgb " +
    String.valueOf(rgb1));
93.                             String rgb2;
94.                             if (rgb1 == -2) {
95.                                 rgb2 = "Click area identification exception";
96.                                 Toast.makeText(getContext(), rgb2,
    Toast.LENGTH_SHORT).show();
97.                             } else {
98.                                 int red2 = (rgb1 & 0xff0000) >> 16;
99.                                 int green2 = (rgb1 & 0x00ff00) >> 8;
100.                                    int blue2 = (rgb1 & 0x0000ff);
101.                                    rgb2 = "选中区域 RGB： " + red2 + " " +
    green2 + " " + blue2;
102.                                    showToast(getFromR(red2));
103.                                }
104.
105.    //                              int pixel = mPic.getPixel((int) event.getX(), (int)
    event.getY());
106.    //                              Log.d(TAG, "pixel " + pixel);
107.    //                              int red = (pixel & 0xff0000) >> 16;
108.    //                              int green = (pixel & 0x00ff00) >> 8;
109.    //                              int blue = (pixel & 0x0000ff);
110.    //                              String rgb = red + " " + green + " " + blue;
111.    //                              Log.d(TAG, rgb);
112.
113.                                invalidate();
114.                                return true;
115.                            }
116.                        } else {
117.                            return false;
118.                        }
```

```
119.                } catch (Exception e) {
120.                    Log.e(TAG, "onTouchEvent: ", e);
121.                }
122.                return false;
123.            }
124.
125.            private String getFromR(int b) {
126.                String a = "-1";
127.                if (b <= 140 && b >= 0) {
128.                    a = "Safe";
129.                }else if (b <= 160 && b > 140) {
130.                    a = "Danger";
131.                }else if (b <= 255 && b > 160) {
132.                    a = "Extreme Danger";
133.                }
134.                if ( "-1".equals(a)) {
135.                    return "The concentration is not within the national detection
    limit.";
136.                }
137.                return String.valueOf(a);
138.            }
139.
140.            private void showToast(String message) {
141.                View                                view                                =
    LayoutInflater.from(getContext()).inflate(R.layout.toast_layout, null);
142.                TextView textView = view.findViewById(R.id.toast_text);
143.                textView.setText(message);
144.                if ("Safe".equals(message)) {
145.                    textView.setTextColor(Color.GREEN);
146.                } else if ("Danger".equals(message)) {
147.                    textView.setTextColor(Color.YELLOW);
148.                }else if ("Extreme Danger".equals(message)) {
149.                    textView.setTextColor(Color.RED);
150.                }
151.                    Toast toast = new Toast(getContext());
152.                toast.setView(view);
153.                toast.setDuration(Toast.LENGTH_SHORT);
154.                toast.setGravity(Gravity.CENTER,0,500);
155.                toast.show();
156.
157.            }
158.
159.    }
```
